# Supplementary material for: Light sources with bias tunable spectrum based on van der Waals interface transistors
Source: Nat Commun. 2022 Jul 7;13:3917. doi: 10.1038/s41467-022-31605-9 (PMC9263129; doi:10.1038/s41467-022-31605-9)
Supplement: Supplementary file 1 — Supplementary Information [file 41467_2022_31605_MOESM1_ESM.pdf]

Supplementary Information: Light Sources with Bias Tunable Spectrum based on van der Waals Interface Transistors

Hugo Henck,[1,2] Diego Mauro,[1,2] Daniil Domaretskiy,[1,2] Marc Philippi,[1,2] Shahriar Memaran,[3,4] Wenkai Zheng,[3,4] Zhengguang Lu,[3,4] Dmitry Shcherbakov,[5] Chun Ning Lau,[5] Dmitry Smirnov,[3,4] Luis Balicas,[3,4] Kenji Watanabe,[6] Takashi Taniguchi,[7] Vladimir I. Fal'ko,[8,9] Ignacio Gutiérrez-Lezama,[1,2] Nicolas Ubrig,[1,2,*] and Alberto F. Morpurgo[1,2,†]

[1]*Department of Quantum Matter Physics, University of Geneva, 24 Quai Ernest Ansermet, CH-1211 Geneva, Switzerland*

[2]*Group of Applied Physics, University of Geneva, 24 Quai Ernest Ansermet, CH-1211 Geneva, Switzerland*

[3]*National High Magnetic Field Laboratory, Tallahassee, FL 32310, USA*

[4]*Department of Physics, Florida State University, Tallahassee, FL 32306-4350, USA*

[5]*Department of Physics, The Ohio State University, Columbus, OH 43210*

[6]*Research Center for Functional Materials, National Institute for Materials Science, 1-1 Namiki, Tsukuba 305-0044, Japan*

[7]*International Center for Materials Nanoarchitectonics, National Institute for Materials Science, 1-1 Namiki, Tsukuba 305-0044, Japan*

[8]*National Graphene Institute, University of Manchester, Booth St E, M13 9PL, Manchester, UK*

[9]*Henry Royce Institute for Advanced Materials, M13 9PL, Manchester, UK*

(Dated: May 23, 2022)

CONTENTS

Supplementary Figures 2
Supplementary Section 1. Different operation regimes of a light emitting field-effect transistor 2
Supplementary Section 2. Output characteristics of 2L MoS_2/5L InSe device 6
Supplementary Section 3. Spectrum of the light emitted by a monolayer TMD LEFET 7
Supplementary References 9

SUPPLEMENTARY FIGURES

Supplementary Section 1. Different operation regimes of a light emitting field-effect transistor

Our work relies on ionic liquid (IL) gated field-effect transistors (FETs) that can emit light when biased in the appropriate regime (*i.e.*, light-emitting field-effect transistors, or LEFETs). Although the use of ionic gating in combination with 2D semiconductors is by now well established, for the convenience of the reader here we provide a brief explanation of the principle of operation of ionic liquid gated FETs (for more details see, for instance, References 1–4).

Whereas in conventional FETs the semiconductor channel is separated from the gate electrode by a solid-state dielectric, an ionic gated transistor exploits an electrolyte to transfer potential from the gate to the surface of the semiconductor. In the devices used in the present work, the gate electrode is a large evaporated Au pad on the substrate supporting the vdW interface contacted by source and drain electrodes (see Figure 1 in the main text). The electrolyte is an IL dropcasted onto the substrate, which covers both the gate electrode and the semiconducting channel (*i.e.*, the vdW interface). Upon the application of a voltage between the gate and drain contacts, the ions in the liquid form two electrical double layers of sub-nanometer thickness at the gate-IL and at the IL-channel interfaces. The electrical

* nicolas.ubrig@unige.ch
† alberto.morpurgo@unige.ch

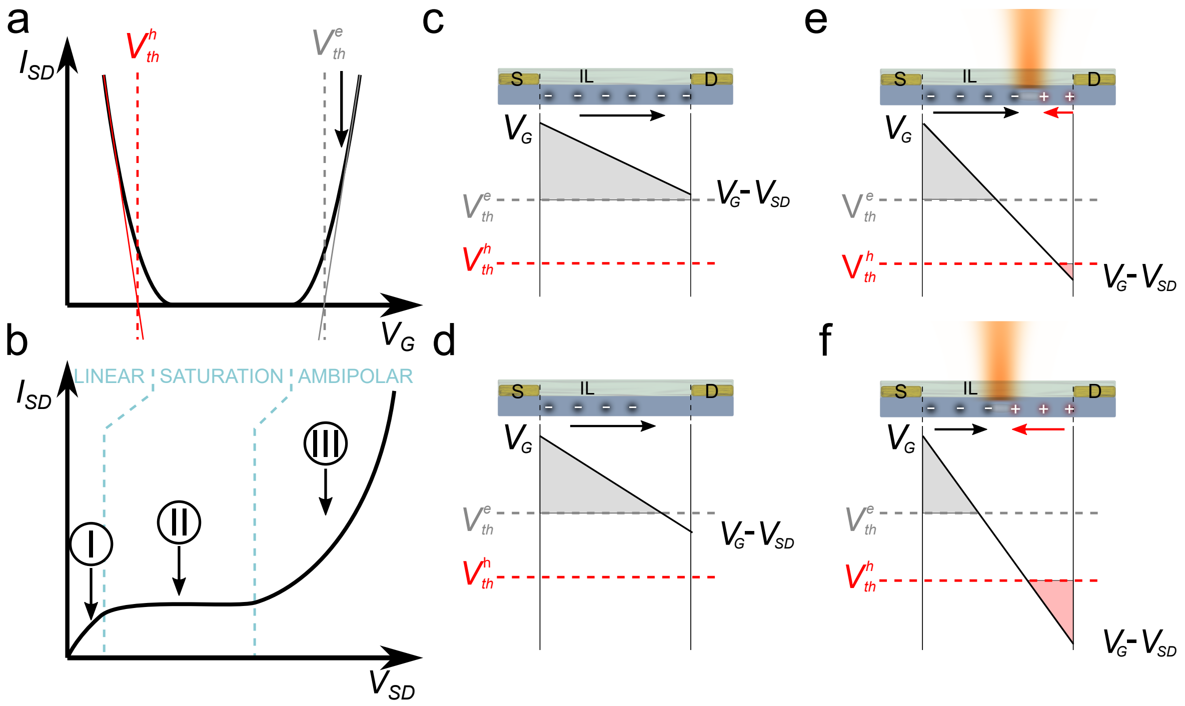

SUPPLEMENTARY FIGURE 1. **Operation regimes of an ionic gated FET.** a Schematic representation of the transfer curve of an ambipolar FET. Threshold voltages for hole and electron conduction are indicated by red and grey dashed vertical lines (they are obtained by extrapolating to zero the $I_{SD}(V_G)$ in the linear regime, as indicated by the continuous lines of the same color). b Schematics of a typical output curve (I_{SD}-vs-V_{SD}) of an ambipolar FET, corresponding to having the applied gate voltage well above threshold (see the vertical arrow in panel a; we consider here the case of electron accumulation). At low bias ($V_{SD} < V_G$-V_{th}^e) the transistor operates in the linear regime (regime I) and the density of electrons varies linearly with position in the transistor channel (as shown in c). As V_{SD} is increased and exceeds V_G-V_{th}^e, the channel is depleted near drain contact (see d) and the source-drain current saturates (regime II in b). Increasing V_{SD} further causes the local potential near the drain to invert and to exceed the threshold for hole accumulation (see e). Under these conditions, the device enters the ambipolar injection regime (regime III in b, in which I_{SD} exhibits an exponential increase) with electrons and holes injected at opposite contacts. Increasing V_{SD} even further leads to a continuous shift of the channel potential, and to a corresponding shift of the position of the *pn*-junction that forms in the channel, where electrons and holes meet (f). Experimentally this shift can be monitored by looking at the position where light due to electron-hole recombination is emitted from (see Supplementary Figure 2).

double layers act as capacitors across which all the applied voltage drops so that the bulk of the IL is equipotential. In practice, the gate area is kept intentionally much larger than the channel area, resulting in a very large capacitance of the gate-IL interface, so that virtually all the voltage applied to the gate drops at the IL-channel interface. The electrical double layers have an extremely large capacitance ($\approx 50\ \mu F/cm^2$) because of their sub-nanometer thickness so that the FETs can be turned on with only a small voltage. As we discuss below, this is key to operate the transistors as LEFETs, because it allows a source-drain voltage much larger than the gate voltage to be applied, as needed to locally invert the potential of the channel at one of the electrodes and to inject simultaneously electrons and holes into the channel.

Conceptually, the principle of operation of an ionic gated FET is identical to that of conventional transistors with solid-state dielectrics. The application of a voltage to the gate allows the accumulation of carriers that –depending on the polarity– can be either electrons or holes. It follows that, for a fixed source-drain bias, the current increases for both positive and negative gate voltages (above the respective thresholds for electron and hole accumulation) resulting in the transfer curve typical of ambipolar transistors, as shown in Supplementary Figure 1a. When the gate is well above threshold (as compared to the applied V_{SD}; see Supplementary Figure 1b), the transistor operates in the linear regime, and in the gradual channel approximation the density of accumulated charge carriers varies linearly with position along the channel, as shown in Supplementary Figure 1c (here we consider the case of electron accumulation; the case of hole accumulation is entirely analogous).

Upon increasing V_{SD} at fixed V_G the local channel potential decreases. Eventually the channel is depleted near the drain contact (Supplementary Figure 1c) and the device enters the saturation regime (Supplementary Figure 1b) just like a conventional FET device. By increasing V_{SD} further, however, the very large capacitance of ionic gated devices allows reaching a regime in which the local potential in the channel near the drain contact inverts and exceeds the threshold for hole accumulation (Supplementary Figure 1e). This is the so-called ambipolar injection regime, in which different types of carriers are injected into the channel at the two contacts (Supplementary Figure 1e; in the case illustrated here electrons

are injected at the source contact and holes are injected at the drain contact). In this regime a *pn*-junction forms in the channel, leading to the exponential increase of the current with increasing V_{SD} (Supplementary Figure 1**b**), as indeed observed in the data shown in the main text. At the location of the *pn*-junction electrons and holes meet in the channel and recombine: if the recombination process is radiative, light is observed and the device acts as a LEFET. Increasing V_{SD} even further causes an increasingly larger shift in the channel potential. The position of the *pn*-junction also shifts (Supplementary Figure 1**f**) accordingly, eventually reaching the source contact. As mentioned in the main text, the process can be easily detected by monitoring the position where light is emitted from, as illustrated by the data measured on our vdW interface LEFETs (see Supplementary Figure 2**a**-**e**).

Both the electrical characteristic of our devices, and the evolution of the light-emission spot with bias indicate that our devices based on vdW Γ-Γ-interfaces exhibit ideal LEFET behavior.[1, 5] We emphasize that conceptually the use of ionic gating is not required to achieve this functionality, and a solid-state dielectric that is sufficiently thin could be used as well, as long as the capacitance and the breakdown voltage are sufficiently large to invert the local potential in the FET channel. Using solid-state dielectric hBN multilayers that are only a few nanometers thick and that have breakdown fields reaching up to 1 V/nm[6], for instance, may enable the realization of LEFETs without the need to employ electrolyte gating. Such three-terminal light-emitting transistors based on hBN dielectric and 2D semiconductors, however, have not yet been demonstrated experimentally, which is why here we resort to using ionic gates (note that light-emitting FETs with solid-state dielectrics have been instead realized using organic semiconductors, whose operation typically requires very large source-drain voltages, allowing the use of much thicker gate dielectrics).

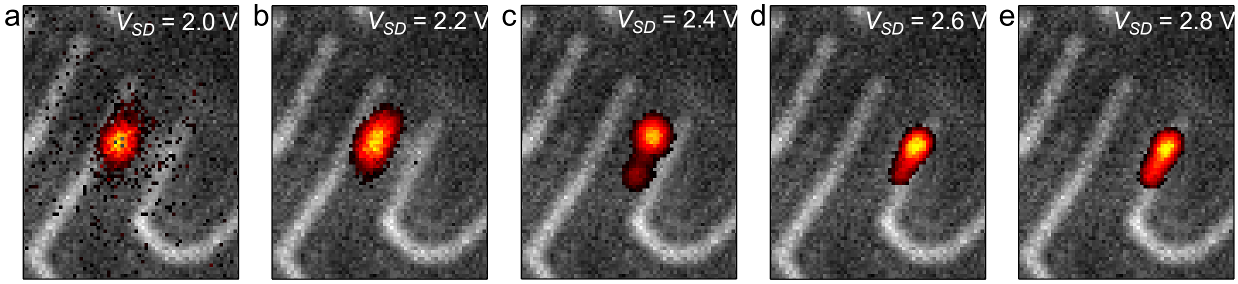

SUPPLEMENTARY FIGURE 2. **Imaging the bias-driven position of the *pn*-junction in a LEFET. a-e** Optical micrographs of the light emitted from a LEFET based on 2L-WS_2/4L-InSe interface at fixed $V_G = +0.3$ V, for V_{SD} increasing from 2.0 V (**a**) to 2.8 V (**e**). As mentioned in the main text and described in Supplementary Section 2, increasing V_{SD} causes the electrostatically-induced *pn*-junction in the transistor channel to shift, resulting in a shift of the bright spot from where light is emitted. The different panels show that the bright spot shifts from the drain to the source contact upon increasing V_{SD}.

Supplementary Section 2. Output characteristics of 2L MoS_2/5L InSe device

In Figure 4**a-e** of the main text, we present the electrical and optical measurements performed on a LEFET based on 2L-MoS_2/5L-InSe. For completeness, here we show (see Supplementary Figure 3**a** and **b**) the output curves (I_{SD}-vs-V_{SD}) of the same device at selected values of V_G, both the electron and hole accumulation mode ($V_G > V_{th}^e$ and $V_G < V_{th}^h$). Similarly to our 2L WS_2/4L InSe device presented in Figure 2**c** and **d** of the main text, we can identify the different regimes characteristic of LEFET operation. Specifically, at low V_{SD} the source-drain current increases linearly until V_{SD} value reaches V_G-V_{th}^e(or V_G-V_{th}^h, when the gate is used to accumulate holes) above which I_{SD} saturates and remains constant (saturation regime). At much larger V_{SD} values, I_{SD} starts increasing steeply, as expected when the device enters the ambipolar injection regime (*i.e.*, the regime in which both types of charge carriers are simultaneously injected into the channel from opposite contacts).

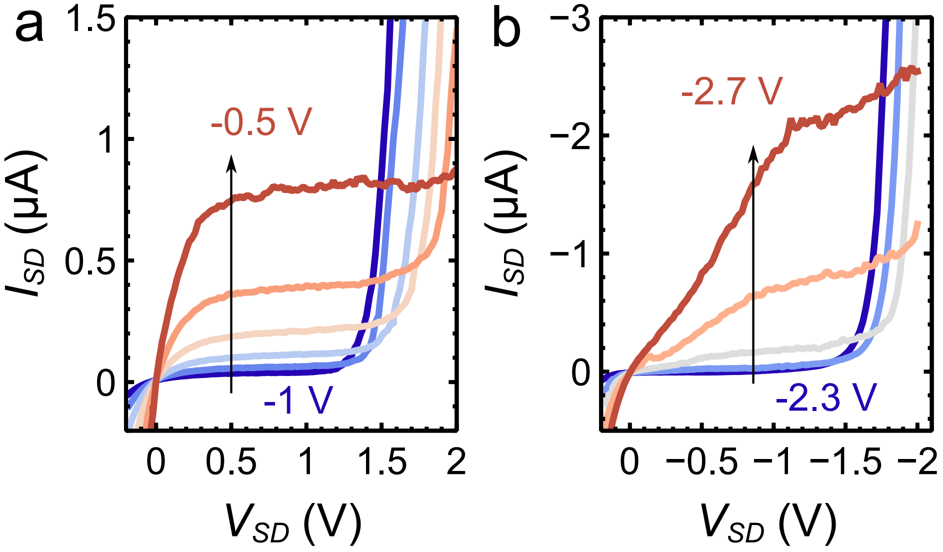

Supplementary Figure 3. **FET output curves for a 2L-MoS$_2$/5L-InSe device. a** and **b**. Output curves at constant V_G of the 2L-MoS$_2$/ 5L-InSe LEFET device, whose data are shown in Figure 4 of the main text. In panel a V_G ranges from -1 V to -0.5 V in 0.1 V steps and in panel b V_G ranges from -2.3 V to -2.7 V in 0.1 V steps.

Supplementary Section 3. Spectrum of the light emitted by a monolayer TMD LEFET

In the introduction of the main text we argued that LEFETs based on vdW interfaces can be expected to allow tuning the spectrum of the emitted light by changing the device operation point because of two main reasons: *i*) in vdW interfaces, interlayer transitions involve states whose energy difference depends on the difference in electrostatic potential between the two layers. The frequency of light emitted by these transitions is therefore expected to shift upon changing the gate voltage, because the electric field perpendicular to the channel (which determines the difference of electrostatic potential between the layers) changes upon changing the gate voltage. *ii*) Thicker multilayers have a rich structure of subbands originating from confinement that –if suitably populated– can participate in radiative transitions at different energy.

These mechanism are not at work in LEFETs based on monolayer TMDs and accordingly we should expect that the spectrum of light emitted by a LEFET realized using a monolayer TMD is virtually insensitive to the device operation point. To verify that this is the case,

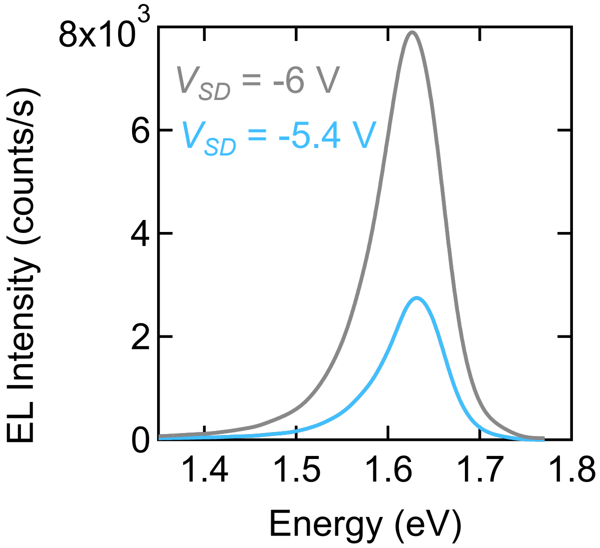

SUPPLEMENTARY FIGURE 4. **Bias insenstivity of the EL spectrum of monolayer LEFETs.** EL spectrum of a LEFET based on monolayer WSe_2. The spectra are recorded under hole accumulation at V_G = -2.5 V. V_{SD} is increased largely above the start of the ambipolar injection regime (grey and blue curves measured respectively at V_{SD} = -6 V and V_{SD} = -5.4 V). The emission does not show any spectral change even at these large V_{SD}-biases, and it virtually matches the spectrum observed in photoluminescence measurements.

we have performed measurements on a LEFET based on monolayer WSe_2 and found that, indeed, the spectrum of the emitted light is always the same irrespective of the values of applied V_G and V_{SD}. This conclusion is illustrated in Supplementary Figure 4 with data measured at negative gate voltage (*i.e.*, with holes that are accumulated at small V_{SD}), in which we see that even when we increase V_{SD} up to very large values, the shape of the spectrum of the emitted light remains virtually unchanged (and corresponds to the spectrum observed in photoluminescence experiments). This observation confirms the notion that LEFETs based on vdW interface have more functionality than the same devices based on monolayers, as they allow the spectrum of the emitted light to be controlled electrically.

SUPPLEMENTARY REFERENCES

[1] M. S. Kang and C. D. Frisbie, A Pedagogical Perspective on Ambipolar FETs, ChemPhysChem **14**, 1547 (2013).

[2] Y. J. Zhang, J. T. Ye, Y. Yomogida, T. Takenobu, and Y. Iwasa, Formation of a Stable p–n Junction in a Liquid-Gated MoS2 Ambipolar Transistor, Nano Letters **13**, 3023 (2013), publisher: American Chemical Society.

[3] S. Z. Bisri, S. Shimizu, M. Nakano, and Y. Iwasa, Endeavor of Iontronics: From Fundamentals to Applications of Ion-Controlled Electronics, Advanced Materials **29**, 1607054 (2017).

[4] I. Gutierrez-Lezama, N. Ubrig, E. Ponomarev, and A. F. Morpurgo, Ionic gate spectroscopy of 2D semiconductors, Nature Reviews Physics **3**, 508 (2021).

[5] J. Zaumseil, R. H. Friend, and H. Sirringhaus, Spatial control of the recombination zone in an ambipolar light-emitting organic transistor, Nature Materials **5**, 69 (2006).

[6] Y. Hattori, T. Taniguchi, K. Watanabe, and K. Nagashio, Layer-by-Layer Dielectric Breakdown of Hexagonal Boron Nitride, ACS Nano **9**, 916 (2015).
